# A review of immunosuppressive therapy in the context of uterine VCA and pregnancy

**DOI:** 10.3389/frtra.2026.1825928

**Published:** 2026-07-06

**Authors:** Andrew D'Elia, Kevin Ghajar, Kevin J. Zuo

**Affiliations:** 1 Division of Plastic, Reconstructive and Aesthetic Surgery, Department of Surgery, University of Toronto, Toronto, ON, Canada; 2 Temerty Faculty of Medicine, University of Toronto, Toronto, ON, Canada; 3 Toronto Western Hospital, University Health Network, Toronto, ON, Canada

**Keywords:** anti-rejection, immune suppression, pregnancy, uterine transplant, uterus, vascular composite allotransplantation

## Abstract

Uterine vascularized composite allotransplantation (VCA) represents one of the latest developments in the field of transplant surgery and treatment of uterine-factor infertility. Unlike other forms of VCA, uterine transplantation involves a temporary allograft, pregnancy-associated physiologic and pharmacokinetic changes, and complex maternal–fetal immunologic interactions, creating distinct challenges in immunosuppression management. Herein, we perform a comprehensive review of the latest and relevant literature on immunosuppressive strategies in the context of VCA and pregnancy, with particular emphasis on their relevance to uterine VCA. Evidence from solid organ transplantation, autoimmune disease, and reported uterine transplant protocols was synthesized to inform stage-specific management strategies. Available data support corticosteroids, calcineurin inhibitors, azathioprine, and hydroxychloroquine as pregnancy-compatible agents, whereas mycophenolate mofetil, methotrexate, and cyclophosphamide remain contraindicated due to teratogenicity. Pregnancy-related pharmacokinetic and pharmacodynamic changes significantly influence drug exposure and necessitate individualized dosing and close therapeutic monitoring. Emerging evidence suggests that pregnancy-associated immune modulation, including regulatory T-cell expansion and microchimerism, may reduce rejection risk, although this remains incompletely characterized. Based on best available evidence, we present a summary for immunosuppressive management spanning induction, preconception optimization, pregnancy, delivery, and postpartum graft removal. Future priorities include development of noninvasive rejection monitoring, precision pharmacokinetic modeling, and tolerance-inducing strategies to improve maternal, fetal, and allograft outcomes in uterine transplantation.

## Graphical Abstract

**Figure:**
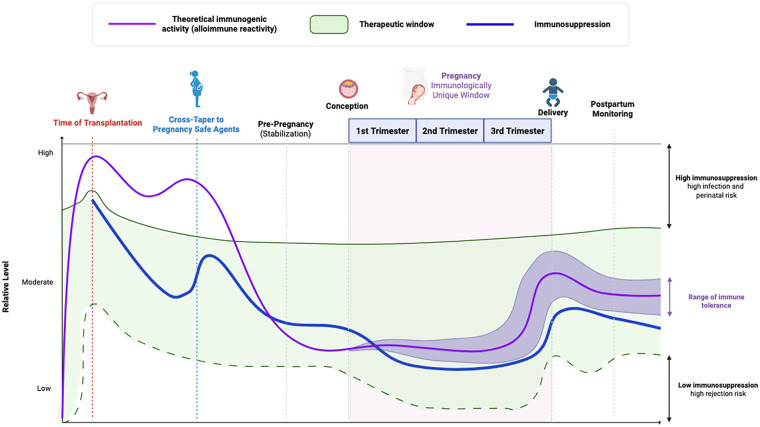
Theoretical immunologic and immunosuppressive activity throughout VCA and pregnancy.

## 1 Introduction

Vascularized composite allotransplantation (VCA) represents one of the most complex reconstructive options in modern reconstructive transplant surgery. To date, VCA has been reported in the reconstruction and restoration of complex anatomy including face, upper extremity, abdominal wall, and uterus (1–3). While strong arguments support the role of VCA in restoring form and function in complex situations, substantial barriers such as ethical considerations, donor availability, and lifelong immunosuppression continue to limit its adoption (1–3).

Uterine VCA challenges traditional immunological and conceptual classification within transplantation. Owing to its later emergence as an innovative technique aimed at improving quality of life, the Organ Procurement and Transplantation Network (OPTN) has classified the uterus as a VCA, despite its closer semblance to a solid organ transplant. Unlike traditional VCA, uterine transplant does not include the transplantation of skin, and thus does not carry with it the same immunogenic burden of these transplants, nor does it present the same ability to monitor for cutaneous and histopathological signs of rejection (4).

Allotransplantation of the uterus and cervix, and occasionally vaginal mucosa, represents a distinct application of VCA aimed at transiently restoring gestational capacity in individuals with uterine-factor infertility who are of reproductive age and desire pregnancy (5). Unlike other forms of VCA, the uterine allograft is temporary, and can be removed following completion of childbearing, thereby eliminating the need for lifelong immunosuppression. However, during the period of graft implantation, recipients require sustained immunosuppression, and this presents the unique double-edged challenge of maintaining allograft survival while safeguarding maternal and fetal health during pregnancy (6–8).

Although the safety of immunosuppressive agents in pregnancy has been partially characterized in the context of autoimmune disease (9, 10), these data are fragmented and not tailored to the physiologic, immunologic, and tissue-specific context of uterine VCA. Differences in uterine immunobiology, the maternal-fetal interface, and pregnancy-associated physiologic changes are well documented and include increased plasma volume, renal clearance, uterine blood flow, hepatic cytochrome (CYP) P450 activity, CYP3A activity, variable changes in CYP2D6, and decreased hematocrit and protein binding (11). Together these factors may alter drug efficacy and toxicity, potentially necessitating alternative dosing strategies or agent selection (11, 12). This is especially important in the context of immune rejection prophylaxis, where maintaining drug serum levels within the therapeutic window is paramount to protect the allograft and the patient from toxicity. As uterine VCA continues to be explored as a therapeutic option for uterine-factor infertility, a better understanding of immunosuppressive safety in this setting is urgently needed.

The literature also suggests that pregnancy invokes a unique immunological state, with several key mechanisms contributing to modulation of the maternal immune system (13). Transplantation, subsequent pregnancy, and the postpartum period thus represent a continuum of immunologic activity with varying immunosuppressive requirements which can be leveraged to maintain graft immune tolerance while minimizing maternal and fetal toxicity.

This review summarizes the available evidence on the use of immunosuppressive agents during pregnancy and contextualizes these findings with immunosuppressive regimens reported in VCA. We aim to identify agents with favorable maternal and fetal safety profiles for use in pregnant uterine VCA recipients, while highlighting additional considerations unique to uterine transplantation and pregnancy physiology that will inform future immunosuppressive protocols.

## 2 Materials and methods

A comprehensive literature search was conducted from database inception through February 2026. The search strategy incorporated combinations of medical subject headings and free-text terms including “uterine transplantation”, “vascularized composite allotransplantation”, “pregnancy”, “immunosuppression”, “maternal–fetal immunology”, “allograft rejection”, “pharmacokinetics”, “immune tolerance”, “transplant pregnancy outcomes” and their synonyms.

Eligible studies included reviews, primary research articles, and meta-analyses published in English that investigated the use of immunosuppressive therapies during pregnancy. Studies examining immunosuppressive strategies in the context of VCA were also included. Exclusion criteria comprised non-English publications and animal studies.

## 3 Results

### 3.1 Safety of immunosuppressive agents in pregnancy

Immunosuppressive therapy is essential during pregnancy in patients with autoimmune disease and solid organ transplantation to preserve graft function and prevent disease flares, with successful pregnancy outcomes achievable when pregnancy-compatible regimens are used (12). In fact, accumulated clinical experience demonstrates that the risks of inadequately controlled disease or graft rejection outweigh the potential pharmacologic risks of appropriately selected immunosuppressive therapy (12, 14). Hence, an understanding of the effects of immunosuppressive agents on both maternal and fetal health during pregnancy is essential for agent selection and can yield insights into strategies amenable to VCA.

#### 3.1.1 Corticosteroids

Corticosteroids are a cornerstone of immunosuppressive therapy during pregnancy, used for maintenance immunosuppression, treatment of rejection, and control of autoimmune disease flares in conditions such as systemic lupus erythematosus (SLE) and vasculitis (10, 15). Prednisone, the most commonly used agent, is hepatically converted to prednisolone, and placental metabolism limits fetal exposure, particularly at daily doses below 20 mg (16).

Across transplant and autoimmune cohorts, corticosteroid exposure has not been associated with an increased risk of major congenital malformations. Maternal complications include gestational hypertension, diabetes, infection, and osteoporosis, while fetal effects, such as transient adrenal suppression, intrauterine growth restriction, and low birth weight are uncommon (10, 15). Prednisone is compatible with breastfeeding, with timing adjustments recommended at higher doses. Corticosteroids are therefore considered safe in pregnancy when used at the lowest effective dose (10, 17, 18).

#### 3.1.2 Calcineurin inhibitors (CNIs)

CNIs, including tacrolimus and cyclosporine, form the backbone of pregnancy-compatible maintenance immunosuppression (19, 20). These agents suppress T-cell activation by inhibiting interleukin-2 (IL-2) signaling and are primarily metabolized through hepatic CYP 3A4 pathways (21–23).

Large registry and cohort studies have not demonstrated an increased risk of major congenital malformations with CNI exposure. However, their use is associated with higher rates of prematurity, low birth weight, and maternal hypertensive disorders (9, 10, 19, 24, 25). Tacrolimus is more frequently associated with maternal diabetes and transient neonatal electrolyte abnormalities, whereas cyclosporine is associated with a higher risk of hypertension and hyperlipidemia (15). Neonatal immune alterations, including transient lymphopenia, have been reported but generally resolve within months (26). Pregnancy-related pharmacokinetic changes often necessitate dose adjustment and close therapeutic drug monitoring to maintain adequate immunosuppression.

Overall, CNIs are considered compatible with pregnancy when appropriately monitored, but they are excreted in breast milk and, thus, breastfeeding is not recommended (10, 17, 18).

#### 3.1.3 Antimetabolites

Azathioprine (AZA) and MMF are among the most widely used and characterized anti-metabolites in immunosuppression regimens but have markedly different safety profiles in pregnancy.

AZA is a purine analog that inhibits lymphocyte proliferation and is commonly used in transplantation and autoimmune disease (27, 28). AZA typically requires treatment for at least 6–8 weeks before reaching full effect (29). Although it crosses the placenta, fetal exposure to active metabolites is limited due to enzymatic immaturity, and no consistent pattern of congenital malformations has been identified (10, 30). Reported risks include prematurity, intrauterine growth restriction, and transient neonatal cytopenias (29). Maternal adverse effects include myelosuppression, and breastfeeding is generally discouraged (26, 29). Overall, AZA is considered the preferred antimetabolite during pregnancy.

In contrast, MMF is a potent inhibitor of lymphocyte proliferation via blockade of inosine monophosphate dehydrogenase. MMF is strongly associated with first-trimester pregnancy loss and congenital malformations, including craniofacial, cardiac, and auditory defects (15, 30). Consequently, MMF is contraindicated in pregnancy and should be discontinued prior to conception, with transition to AZA when ongoing antimetabolite therapy is necessary (9, 17, 18, 31, 32).

#### 3.1.4 Alkylating agents

Cyclophosphamide is an alkylating agent used in oncology and severe autoimmune disease, exerting its immunosuppressive effects through DNA crosslinking and inhibition of cellular proliferation, particularly in rapidly dividing lymphocytes (142–144).

Its use in pregnancy is limited by significant teratogenic risk. First trimester exposure is associated with severe fetal malformations, collectively described as “cyclophosphamide embryopathy”, including growth restriction, craniofacial abnormalities, and limb defects. In contrast, reports of second- and third-trimester exposure in the context of maternal malignancy or autoimmune disease have not consistently demonstrated an increased risk of congenital malformations, although data remain limited and should be interpreted cautiously (145–153).

Cyclophosphamide is excreted in breast milk, and while reported infant exposure is low, cases of neonatal neutropenia have been described; therefore, breastfeeding is generally avoided during treatment (154). Additionally, cyclophosphamide is associated with gonadotoxicity and may impair future fertility (155, 156).

Given these risks, cyclophosphamide is generally contraindicated in pregnancy, particularly during the first trimester, and should be reserved for life-threatening maternal conditions where no safer alternatives exist.

#### 3.1.5 Immunomodulatory agents

Hydroxychloroquine is an immunomodulatory agent commonly used in SLE and antiphospholipid syndrome (APS). It exerts its effects by disrupting antigen processing and presentation and inhibiting toll-like receptor signaling, thereby reducing T-cell activation and pro-inflammatory cytokine production (10, 157, 158). Hydroxychloroquine is hepatically metabolized by CYP P450, with renal excretion of its metabolites (159).

In pregnancy, although volume of distribution increases, drug clearance remains unchanged, and standard dosing (≤400 mg/day) is appropriate (160). Continued use is recommended, as hydroxychloroquine has been shown to reduce maternal disease flares and improve pregnancy outcomes without increasing the risk of congenital malformations or adverse long-term neurodevelopmental effects (17). Although it crosses the placenta and is excreted in breast milk, available evidence supports its safety during breastfeeding (161, 162).

In summary, pregnancy-compatible agents include corticosteroids, CNIs (tacrolimus, cyclosporine), AZA, and hydroxychloroquine, which, according to available evidence, do not increase major congenital malformations above baseline risk, though they are associated with higher rates of prematurity, low birth weight, and maternal complications such as hypertension and diabetes (Table 1) (24, 33). In contrast, MMF, methotrexate, leflunomide, and cyclophosphamide are contraindicated due to established gonadotoxicity and teratogenicity in the first trimester, requiring discontinuation prior to conception with transition to safer alternatives (e.g., switch MMF to AZA) (17, 19, 24, 30, 33–35).

**Table 1:** Immunosuppressants in pregnancy.

Drug	Pregnancy compatibility	Key maternal risks	Key fetal/neonatal risks	Clinical action
Corticosteroids	Compatible	GDM, HTN, infection, osteoporosis	Rare adrenal suppression; possible IUGR at high dose	Use lowest effective dose
Tacrolimus	Compatible (preferred CNI)	HTN, nephrotoxicity, GDM	Prematurity, LBW, transient electrolyte disturbance	Monitor free drug if available; allow 3–5 ng/mL in stable pregnancy
Cyclosporine	Compatible	HTN, hyperlipidemia, nephrotoxicity	Prematurity, LBW	Use only if tacrolimus intolerant
Azathioprine	Compatible (preferred antimetabolite)	Cytopenias, hepatotoxicity	Prematurity, IUGR, transient cytopenias	Substitute for MMF ≥6 weeks pre-conception
MMF	CONTRAINDICATED	Cytopenias	First-trimester loss; craniofacial, cardiac, limb defects	Discontinue ≥6 weeks before conception
Cyclophosphamide	CONTRAINDICATED	Gonadotoxicity, myelosuppression	Cyclophosphamide embryopathy	Avoid; not appropriate for uterine VCA
Hydroxychloroquine	Compatible	Rare retinal toxicity	No increased malformations	Continue at standard dose if indicated

AZA, azathioprine; MMF, mycophenylate mofetil; SLE, systemic lupus erythematosus; APS, antiphospholipid antibody syndrome; HTN, hypertension; DM, diabetes mellitus; GI, gastrointestinal; IUGR, intrauterine growth restriction; BP, blood pressure.

### 3.2 Pharmacokinetic and pharmacodynamic changes in pregnancy

#### 3.2.1 Volume of distribution

Pregnancy increases the Vd for most drugs through multiple physiological mechanisms (36). Throughout pregnancy, maternal plasma volume increases and peaks by 40%–50% in the third trimester, a mechanism mediated by estrogen's activation of the renin-angiotensin-aldosterone system (37, 38). Pregnancy also increases average total body water by 8 liters, thereby increasing the Vd for hydrophilic drugs and, thus, decreasing their peak serum concentrations. Lastly, increased maternal fat stores during pregnancy increase the Vd for lipophilic drugs (39). Together, increased volumes of distribution decrease the initial and peak concentrations of different drugs, necessitating revision drug dosing and therapeutic monitoring to maintain efficacy and prevent toxicity and allograft rejection (12, 40).

#### 3.2.2 Protein binding and hypoalbuminemia

The increase in plasma volume during pregnancy decreases serum albumin levels which directly impacts the concentration of drugs, including immunosuppressants like tacrolimus, which are highly protein bound (41). Clinical interpretation of hypoalbuminemia is complex and dependent on properties of the drug in question including the fraction of it that would become free (unbound). For tacrolimus specifically, therapeutic drug monitoring typically measures total whole-blood concentrations, which may appear subtherapeutic during pregnancy despite adequate free drug concentrations (42). This can lead to unnecessary dose escalations when targeting standard whole-blood trough levels, potentially resulting in elevated unbound concentrations and toxicity.

#### 3.2.3 Placental metabolism

In addition to immune modulation, the placenta confers significant metabolic activity, with demonstrated CYP isoform expression, glucuronidase activity, and other enzymes at the maternal-fetal interface, which may contribute to metabolism of various agents including immunosuppressants. The metabolic activity of the placenta is also thought to regulate hormone signaling and neutralize xenobiotics throughout gestation (43). While the placenta exhibits metabolic activity, this activity is generally quite narrow, localized, and limited in effect relative to other host mechanisms of metabolism (44). In a similar vein, there are no reports of appreciable changes in metabolism outside of the fetal circulation attributed to placenta. From this perspective, there remains space for more work to evaluate the influence that placental metabolism has on the pharmacokinetics of patients' immunosuppressive therapy.

#### 3.2.4 CYP induction and drug clearance

Pregnancy induces differential changes in CYP P450 enzyme activity, with important implications for immunosuppressive drug metabolism. The most clinically relevant change is the induction of CYP3A4 activity, which increases progressively throughout pregnancy, reaching 1.25-fold, 1.75-fold, and 2.32-fold of non-pregnant levels by the end of the first, second, and third trimesters, respectively (45).

Tacrolimus is primarily metabolized by CYP3A4 and CYP3A5, so enzyme induction significantly increases tacrolimus clearance during pregnancy (12). Specifically, tacrolimus exhibits significant trough level variability during pregnancy, with concentration-to-dose ratios decreasing by approximately 48%–61% compared to pre-pregnancy levels (46, 47). In turn, dose increases to maintain therapeutic trough concentrations and prevent allograft rejection is necessary.

Ultimately, the different pharmacokinetic and pharmacodynamic changes during pregnancy necessitate careful monitoring of drug concentrations to protect the allograft while mitigating maternal and fetal toxicity.

### 3.3 Unique immunology of uterine transplantation and pregnancy

Pregnancy is characterized by a distinct immunologic state in which the maternal immune system adapts to tolerate semi-allogeneic fetal tissue. In contrast to the typical immune response mounted against foreign antigens, fetal cells expressing paternal alloantigens evade rejection through tightly regulated mechanisms of immune modulation and tolerance at the maternal–fetal interface.

In VCA, this potentially tolerogenic environment is preceded by the transplantation process itself, which, in the context of surgery, represents an acutely elevated immunologic state. Uterine transplantation is accompanied by major surgical trauma, ischemia-reperfusion injury, and early immune cell trafficking into the allograft, together producing an acutely inflammatory and immunogenic postoperative milieu (48). Although this early inflammatory phase generally resolves within the first 2 weeks after transplantation, it likely contributes to the heightened risk of rejection observed early after graft implantation and underscores the need for robust induction and maintenance immunosuppression during this period (49).

Accordingly, the immunology of uterine VCA is best understood as dynamic and stage-dependent. In the immediate post-transplant period, inflammatory injury and alloimmune activation predominate, whereas during pregnancy, placental and maternal immune adaptations may shift the balance toward relative tolerance (50). The extent to which pregnancy-related immune modulation truly protects the uterine allograft remains uncertain, but it represents an important area for future investigation with potential implications for rejection surveillance, dosing intensity, and selection of maintenance and anti-rejection therapies in uterine VCA recipients who become pregnant.

#### 3.3.1 T-cell modulation

The multifaceted role of the placenta in pregnancy extends to both maternal and fetal immune function. Interestingly, the placenta signals the inhibition of natural killer cells, augments anti-inflammatory and regulatory T-lymphocytes (Tregs), and even suppresses and/or induces programmed cell death in pro-inflammatory T-cells (51–53). Studies have uncovered placental epigenetic silencing of HLA polymorphisms and various chemokines (52, 54–56). Tregs at the maternal-fetal interface promote tolerance by secreting IL-10 and transforming growth factor beta, which suppress alloreactive and self-reactive lymphocytes. The immunologic activity of the placenta has been observed both within the local microenvironment of the maternal-fetal interface as well as the maternal circulation. These pathways play a critical role in maternal tolerance of paternal antigens throughout and even after gestation, safeguarding mother and baby from immune reactivity (57). In the setting of uterine VCA, these pregnancy-associated immune adaptations may theoretically confer a degree of immune privilege to the grafted uterus (58).

#### 3.3.2 Microchimerism

Fetal microchimerism refers to the presence of genetically distinct fetal cells in the mother, acquired through bidirectional transplacental trafficking during pregnancy (59). These cells, often CD34+ hematopoietic progenitors or immune cells, can integrate into various maternal tissues, including thyroid, liver, cervix, and hematopoietic organs, and persist in them for decades following parturition (60).

Microchimerism can impart bimodal immunologic effects. On one hand, tolerogenic effects include expansion of fetal antigen-specific Tregs and induction of split tolerance via extracellular vesicle-mediated dendritic cell modification (61). On the other hand, increased microchimerism has been implicated in autoimmune diseases such as scleroderma and SLE (59, 60). Fetal cells also participate in maternal tissue repair (62). The mechanisms by which these distinct immunologic phenotypes of microchimerism develop remains under investigation.

Evidence from transplantation further illustrates this duality. Although microchimerism has been proposed as a potential contributor to immune tolerance, it does not appear to consistently protect against donor-specific antibody formation or graft injury (63). In some settings, higher levels of donor-specific microchimerism have instead been associated with ongoing alloimmune activation and graft dysfunction. In kidney transplantation, detectable microchimerism has been reported in 75% of patients with allograft dysfunction compared with 29% of those with stable graft function, with significantly higher mean donor genome equivalents observed in the dysfunction group (64). Similarly, in liver transplantation, microchimerism has been detected in 72% of recipients and has been significantly associated with rejection episodes (65).

Donor-specific antibody formation is unlikely to be driven directly by microchimerism itself, and may instead reflect mechanisms such as inverted direct allorecognition, microchimerism may still serve as a marker of persistent alloantigen exposure (66). Overall, the biological role of microchimerism remains controversial, with evidence supporting its association with both immune tolerance and immune activation (13). As such, its immunologic significance appears to be highly context-dependent, varying according to the degree of chimerism, organ type, timing after transplantation, and the surrounding immune milieu.

#### 3.3.3 Potential protective effects of pregnancy on rejection

In solid organ transplant recipients with stable graft function prior to conception, pregnancy is not associated with an increase in graft loss or rejection (12, 67). The incidence of acute allograft rejection during pregnancy is approximately 2.4%, and graft loss during pregnancy is approximately 1.6%, which is comparable to background rejection rates in nonpregnant transplant recipients (67).

In uterine VCA recipients, pregnancy may reduce rejection risk (68, 69). Specifically, rejection episodes are more frequent in the early post-transplant period (44% during months 1–5) than during pregnancy (28% during months 6–10). In the Dallas Uterus Transplant Study, only one rejection episode was detected during pregnancy among 11 recipients who achieved live births, which resolved with steroid treatment (70). The ISUTx registry reports that most rejection episodes occur before pregnancy attempts, with lower rates during gestation (68). No studies have directly compared rejection rates during pregnancy vs. non-pregnant periods in uterine VCA recipients. Whether pregnancy-induced tolerance mechanisms (Treg expansion, hormonal immunomodulation) contribute to reduced rejection risk during gestation in uterine VCA remains speculative and represents an important area for future research.

### 3.4 Current immunosuppressive strategies in VCA and uterine transplant

The first attempt at VCA transplantation was made in 1964 with the technically successful transplantation of a hand under AZA and prednisone, but it unfortunately failed due to acute rejection only a few weeks later (71). Rates of rejection in VCA are high, with acute rejection occurring in about 80% of hand and face transplant recipients in the first postoperative year (72). Chronic rejection, while less characterized, can also lead to graft vasculopathy and potential loss (73). Hence, immunosuppression is essential for preventing rejection in VCA, and it is typically divided into three distinct regimens.

The nascency of reproduction facilitated via uterine VCA means that current techniques remain experimental, and available evidence on outcomes is low quality (74). Thus, current understanding of the effects of immunosuppression on adverse events in uterine transplant pregnancy remains poor. A systematic review of 40 live uterine transplant pregnancies reported high rates of perinatal prematurity (70%), respiratory distress (35%), and birth weight less than the 10th percentile (20%) (74). While the etiology of prematurity in uterine transplant pregnancies is unclear, prematurity is similarly reported in solid-organ transplant recipients and may represent underlying contributions from immunosuppression (75, 76). Pre-eclampsia was documented in 15% of cases, premature rupture of membranes in 15% of cases, and gestational diabetes was documented in 7.5% of cases. Unfortunately, consideration of immunosuppressive regimen was not included in this analysis.

#### 3.4.1 Induction

Induction therapy is typically administered perioperatively to reduce the risk of early acute rejection. Protocols are largely adopted from those in solid organ transplantation and typically involve lymphocyte-depleting agents such as anti-thymocyte globulin (ATG) and alemtuzumab (anti-CD52 agent) or IL-2-receptor antagonists (basiliximab) (72, 77, 78).

ATG is a polyclonal antibody preparation that exerts its immunosuppressive effect by depleting T- and B-cells using complement-dependent lysis and apoptosis, interfering with dendritic cell function, and expanding the repertoire of Tregs (79). In VCA protocols, ATG is typically administered intravenously (1.5 mg/kg daily for 3–5 doses) often part of a multi-agent induction strategy with agents such as CNIs, corticosteroids, and antimetabolites to promote tolerance and minimize long-term immunosuppression (80, 81). ATG is also considered in VCA recipients with high immunologic risk or in desensitization protocols due to its lymphocyte-depleting capacity (82). Monitoring of CD3+ T-cell counts is used to guide dosing, and infection prophylaxis is recommended due to the risk of leukopenia and immunosuppression (81).

Alemtuzumab is an anti-CD52 humanized monoclonal antibody that decreases the risk of acute rejection by leading to a rapid and sustained reduction of circulating B- and T-cells through antibody-mediated cellular cytotoxicity and complement-mediated lysis (83). It also has immunomodulatory effects that promote graft survival by expanding the pool of regulatory B-cell (84). Basiliximab is a chimeric monoclonal antibody that binds specifically to the alpha subunit of the IL-2 receptor on activated T-lymphocytes (85). Unlike alemtuzumab, basiliximab does not deplete lymphocytes, but instead is a selective inhibitor of IL-2-mediated T-cell activation. This more targeted approach is associated with a lower risk of infection and other adverse events but may not be as effective in preventing acute rejection in highly immunogenic VCA tissues.

Although both are used as induction agents, alemtuzumab is favored for its potent lymphocyte depletion and basiliximab for its safety profile and selective immunosuppression. The choice depends on recipient risk factors and center-specific protocols.

Uterine transplantation presents a unique immunologic interface in VCA, but this has yet to be explored in the context of uterine VCA-specific induction regimens. The Swedish protocol, cited in the first case report of livebirth following uterine transplant, was formulated based on local guidelines for kidney transplant (86, 87). The protocol used intravenous ATG (1.5–2.5 mg/kg) and methylprednisolone (500–1,000 mg) as induction therapy prior to surgery. The use of intravenous ATG and corticosteroids like methylprednisolone as induction agents is consistently reported in multicenter registry data and large clinical series such as the International Society of Uterus Transplantation, the Dallas Uterus Transplant Study and the US Uterus Transplant Consortium (7, 68, 88).

#### 3.4.2 Maintenance

Maintenance therapy is essential for long-term graft survival in VCA and generally consists of a combination of a CNI (typically tacrolimus), an antimetabolite (MMF), and corticosteroids (prednisone) (89–91). This triple-drug regimen is the most widely used approach and is adapted from protocols established in solid organ transplantation while accounting for the high immunogenicity of multiple tissues types in VCA, particularly skin (92). Tacrolimus is typically dosed to achieve target trough levels of 6–12 ng/mL in the early post-transplant period with gradual reduction over time. MMF is administered at a dose of 1–2 g a day and prednisone is tapered to the lowest effective dose, often 5–10 mg daily (89, 93).

Efforts to minimize steroid exposure and systemic toxicity are ongoing. Steroid withdrawal protocols and dual-agent regimens (tacrolimus plus MMF) have been implemented to reduce adverse effects while maintaining efficacy, with clinical series demonstrating feasibility and acceptable rejection rates when steroids are weaned after the early post-transplant period (89, 93).

Local immunosuppressive maintenance strategies, such as topical tacrolimus and mycophenolic acid, are being investigated to further reduce systemic toxicity while sustaining graft survival and these remain experimental (90, 94).

Current regimens for maintenance immunosuppression in uterine differ from standard VCA regimens. Tacrolimus is dosed to achieve target trough levels of 8–10 ng/mL in the first 3 months post-transplant then reduced to 3–5 ng/mL by one year, with these targets maintained during pregnancy. MMF is used for the first 6–10 months post-transplant but is then replaced by AZA prior to embryo transfer and during pregnancy due to MMF's teratogenicity concerns (88). Prednisone or prednisolone is often included at 5–10 mg daily, with some protocols tapering or discontinuing steroids after the early post-transplant period.

#### 3.4.3 Rejection management

Rejection management involves an escalation of immunosuppression in response to biopsy-proven rejection episodes. For Banff rejection grades I and II, first-line agents to treat acute rejection episodes incorporate high-dose systemic corticosteroids, typically intravenous methylprednisolone, topical corticosteroids (e.g., clobetasol), and topical tacrolimus for cutaneous manifestations (95).

In cases of steroid-resistant or severe rejection (Banff grades III and IV), rejection management includes ATG, alemtuzumab, rituximab, and, less commonly, basiliximab or sirolimus. ATG is given intravenously, typically at 1.5 mg/kg daily for 3–5 days, while alemtuzumab is used as a single dose (30 mg intravenously), and rituximab is reserved for antibody-mediated or B-cell–driven rejection (95). Plasmapheresis and intravenous immunoglobulin (IVIG) are considered in cases of antibody-mediated rejection, though this is rare in VCA.

Adjunctive strategies include temporary augmentation of maintenance immunosuppression (increasing tacrolimus or MMF dosing), and local drug delivery systems for tacrolimus, which are under investigation to reduce systemic toxicity (90).

Regimens for treating rejection episodes are similar in uterine VCA. For borderline or mild episodes, observation with repeat biopsy or an increase in baseline immunosuppression (higher tacrolimus or introduction of oral steroids) is recommended (6, 48). Moderate and severe rejection episodes are managed with a 3-day course of intravenous methylprednisolone (day 1: 1,000 mg; days 2–3: 500 mg), followed by oral steroid taper (6, 88, 96). For steroid-resistant cases, 1–1.5 mg/kg per dose of ATG is administered intravenously for 3–5 days.

In rare cases of antibody-mediated rejection, multimodal therapy may include ATG, plasmapheresis, IVIG, and increased maintenance immunosuppression (higher tacrolimus trough, reintroduction of MMF, and increased prednisone) (88).

Acute rejection during pregnancy has been documented in uterine VCA using cervical punch biopsies performed at 12–14 and 24–26 weeks gestation for surveillance (6, 88). Reported rejection was successfully managed with temporary increases in steroid dosing or CNI adjustment, and most resolved without adverse impact on pregnancy outcomes.

The immunosuppressive and immunomodulatory agents utilized in uterine VCA protocols are summarized in Table 2.

**Table 2:** Immunosuppressants in uterine VCA.

Drug	Phase	Uterine VCA dosing	Uterus-specific rationale
ATG	Induction	1.5–2.5 mg/kg IV × 3–5 doses, first pre-reperfusion	Aggressive depletion justified by short reproductive window
Methylprednisolone	Induction; rejection	500–1,000 mg IV at transplant; 1,000 mg × 1 then 500 mg × 2 for moderate-severe rejection	Standard
Tacrolimus	Maintenance	Trough 8–10 ng/mL early; 3–5 ng/mL late and during stable pregnancy	Allow lower trough during stable pregnancy given pregnancy-induced tolerance and free-drug elevation
AZA	Maintenance during pregnancy	Standard dosing; substitute for MMF ≥6 weeks pre-conception	MMF teratogenic; AZA acceptable fetal risk
MMF	Pre-conception only	1–2 g/day; discontinue ≥6 weeks before embryo transfer	Avoid throughout pregnancy
Prednisone	Maintenance	5–10 mg PO daily	Lowest effective dose to minimize GDM/HTN
Hydroxychloroquine	Adjunct (autoimmune overlap)	≤400 mg/day	Continue if indicated; pregnancy-safe

ATG, anti-thymocyte globulin; AZA, azathioprine; CNI, calcineurin inhibitor; GDM, gestational diabetes mellitus; HTN, hypertension; IUGR, intrauterine growth restriction; LBW, low birth weight; MMF, mycophenolate mofetil; VCA, vascularized composite allotransplantation.

#### 3.4.4 Infection and prophylaxis

These potent immunosuppression regimens, required for allograft survival, come at the elevated risk of bacterial, viral, and fungal infections (97–100). In uterine VCA, infection is a common yet manageable complication with minimal impact on graft or pregnancy outcomes (6, 101). Urinary tract infections are the most commonly reported, while CMV viremia and COVID-19 were also reported. Due to the potential burden of these infections on graft function and hence maternal and fetal health, infection prophylaxis is necessary. Protocols usually consist of pre-transplant infectious disease screening, vaccination, and individualized counseling for transplant recipients planning pregnancy.

CMV screening of both donor and recipient is standard, and antiviral prophylaxis or preemptive treatment for 3–6 months post-transplant is consensus practice to reduce CMV-related morbidity (12). For bacterial prophylaxis, perioperative antibiotics (piperacillin-tazobactam, cefazolin, and amoxicillin-clavulanic acid) are administered, and urinary tract infection is managed with prompt diagnosis and treatment (87, 88, 102). COVID-19 vaccination is recommended, and breakthrough infections have not been associated with adverse pregnancy outcomes in uterus transplant recipients.

### 3.5 Additional considerations for the pregnant VCA patient

#### 3.5.1 Fertility and IVF outcomes

Although there are no published studies specifically addressing the effects of immunosuppression on fertility in pregnant VCA patients, the consensus is that immunosuppression can impact fertility and pregnancy outcomes. Principles for VCA recipients seeking pregnancy should thus be taken from best evidence arising from solid organ transplantation and weighed against data from studies on immunosuppression in pregnancy.

Certain immunosuppressants can impair gonadal function and reduce fertility, particularly with prolonged use or exposure to alkylating agents like cyclophosphamide. Thus, pregnancy should be planned, and immunosuppressive regimens should be optimized prior to conception to minimize risks to both mother and fetus (76). Tacrolimus has also been associated with above-baseline rates of infertility in renal transplant recipients but interestingly has otherwise been described as a treatment for unexplained/immune female infertility quite broadly (103–106). The effect of MMF on fertility is not adequately described, and most reports remain based on animal models (103). In the context of uterine transplant, immunosuppression may therefore theoretically interfere with successful implantation and/or initiation of pregnancy.

In addition, the effect of abovementioned immunosuppressive agents on the success of *in vitro* fertilization (IVF), standard in the case of uterine transplant, is poorly understood. In a retrospective study of 37 women with SLE/APS, IVF was safely and successfully performed despite receiving therapy with hydroxychloroquine (72%), steroids (70%) or AZA (3%), with an average of 2.6 cycles of IVF required per patient (107). A case report from a renal transplant patient on AZA, cyclosporine and methylprednisolone reported successful singleton pregnancy via IVF after three failed trials of artificial insemination (108). More work is needed in this context to guide discussion, planning and treatment around other aspects of fertility care.

## 4 Discussion

### 4.1 Pregnancy-specific management in uterine VCA

Available evidence highlights the complex interplay between transplant immunobiology, pregnancy physiology, and pharmacologic safety in uterine VCA.

Importantly, pregnancy itself should not be viewed solely as a physiologic state requiring modification of immunosuppressive regimens based on pharmacodynamic shifts, but rather as an active immunologic modifier of graft tolerance (12). The maternal immune system undergoes a coordinated shift toward a tolerogenic profile, characterized by expansion of Tregs, suppression of pro-inflammatory T-cell responses, and modulation of innate immune activity at both the maternal–fetal interface and systemic level (109, 110). In parallel, bidirectional trafficking of fetal and maternal cells results in microchimerism, a phenomenon that has been implicated in long-term immune adaptation through mechanisms such as antigen-specific Treg expansion and dendritic cell reprogramming (111), with implications in chronic low grade alloimmune rejection and donor-specific antibody formation as well.

Collectively, these processes may promote a state of partial immune tolerance that extends beyond fetal protection and may influence allograft recognition and rejection dynamics. Emerging clinical observations, including lower rates of acute allograft rejection during pregnancy in uterine VCA recipients, support this hypothesis (68, 69). Framing pregnancy as an endogenous immunomodulatory state has important implications for immunosuppressive strategy, raising the possibility that physiologic tolerance mechanisms could be synergized with controlled immunosuppression to reduce toxicity, and inform future tolerance-inducing approaches in uterine transplantation.

Despite this, current immunosuppressive regimens in uterine VCA remain largely extrapolated from kidney and other solid organ transplantation protocols, without full consideration of the unique milieu of uterine transplantation. Goals of immunosuppression in solid organ transplant, including long-term graft survival and attenuation of a fully competent immune system, however, do not apply to uterine VCA. In contrast to permanent solid organ grafts, uterine VCA involves a temporary allograft that directly participates in pregnancy, requiring a dynamic balance between graft protection and fetal safety across distinct clinical stages.

Accordingly, immunosuppressive management should be conceptualized as a uterus-specific, stage-dependent strategy that is adapted to the patient's unique tolerogenic or immunogenic response to pregnancy. In the early post-transplant period, robust immunosuppression is required to mitigate the high risk of acute rejection characteristic of VCA. As patients transition toward conception, regimens should be optimized toward pregnancy-compatible agents, including proactive substitution of teratogenic drugs such as MMF. During pregnancy, management should emphasize close therapeutic monitoring and may account for endogenous tolerance mechanisms, including Treg expansion and microchimerism, which can plausibly lower rejection risk. Precise monitoring control of immunosuppressive therapy is paramount in facilitating an immunologically tolerant phenotype of pregnancy and avoiding immunogenic states. Finally, the planned removal of the graft following completion of childbearing provides a unique opportunity to minimize long-term immunosuppressive exposure. These ideas are further explored in the following subsections.

**Figure 1:**
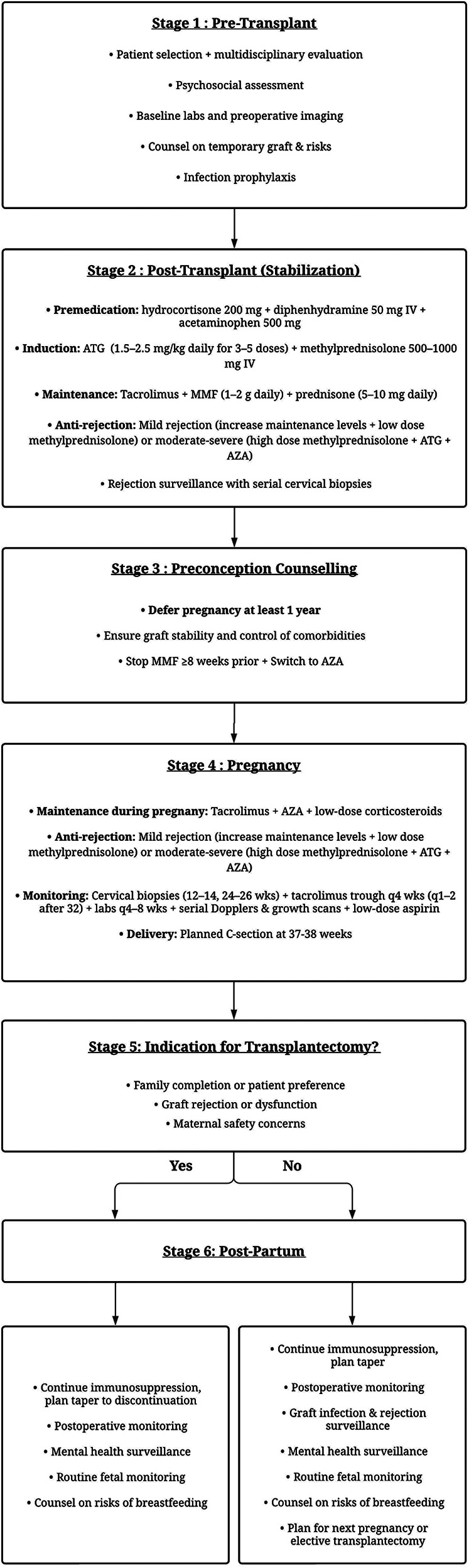
Proposed algorithm for immunosuppression management in uterine VCA across the reproductive timeline. Attached per instructions.

Taken together, these considerations support a shift away from direct extrapolation of solid organ transplant protocols toward a tailored, uterus-specific framework for immunosuppression, as outlined in Figure 1.

#### 4.1.1 Induction

Induction therapy in uterine VCA must balance two competing priorities: minimizing early acute rejection while preserving maternal safety and future reproductive potential. Induction regimens should avoid agents associated with long-term gonadotoxicity or teratogenicity while maintaining adequate lymphocyte depletion to prevent early graft loss.

Based on our review, ATG combined with high-dose corticosteroids appears to represent the most appropriate induction strategy in uterine VCA. The Swedish and U.S. protocols have consistently utilized intravenous ATG at 1.5–2.5 mg/kg daily for 3–5 doses, initiated perioperatively prior to graft reperfusion, in combination with methylprednisolone 500–1,000 mg intravenously at the time of transplantation, followed by tapering (7, 68, 88). Other studies further specify that the first administration of ATG should occur before graft reperfusion and be repeated on postoperative days 2 and 4. The regimen, however, should be preceded by premedication with hydrocortisone 200 mg, diphenhydramine 50 mg IV and acetaminophen 500 mg to prevent common adverse reactions. Close monitoring of blood cell and platelet count is also important in the early postoperative period because doses are adjusted for thrombocytopenia: if the platelet count is <25,000  × 106/L the dose will be cut in half; if the platelet count is <15,000 × 106/L, the dose will be held for 24 h, and the platelet count will be reassessed (8).

#### 4.1.2 Maintenance

##### 4.1.2.1 Prior to pregnancy

In the immediate and intermediate post-transplant period, uterine VCA recipients require robust maintenance immunosuppression to prevent acute allograft rejection, especially in the context of an acute inflammatory/postoperative state. Most protocols mirror solid-organ transplantation and employ triple therapy consisting of a CNI, an antimetabolite, and corticosteroids.

During this phase, tacrolimus-based regimens are generally preferred, given their widespread use in transplant pregnancy cohorts and favorable comparative profile relative to cyclosporine (lower rates of hypertension and hyperlipidemia). Tacrolimus is typically dosed to maintain trough levels of approximately 8–10 ng/mL during the first 3 months post-transplant, with gradual reduction thereafter. Low-dose prednisone (5–10 mg daily) is often included, with some centers implementing steroid minimization protocols once graft stability is achieved (89–91). An antimetabolite, most commonly MMF at 1–2 g daily, is frequently used in the early post-transplant period due to its superior efficacy in preventing acute rejection compared to AZA.

Current clinical practice supports transitioning from MMF to AZA prior to planned pregnancy, typically allowing a washout period of at least 6 weeks before embryo transfer (9, 32). Reports in uterine VCA typically achieve maintenance immunosuppression with MMF for the first 8 months, followed by transition to AZA for two months prior to attempted pregnancy. Other studies successfully report *de novo* maintenance therapy with AZA, eliminating the indication for cross-tapering of antimetabolite therapy. Beyond the known teratogenicity of MMF, the latter approach may be preferable as cross-tapering poses a theoretical risk of destabilized immunosuppression leading to graft rejection.

With that being said, the optimal maintenance regimen has not yet been established and is primarily based on experience in kidney transplantation (8). Our synthesis is nonetheless consistent with regimens reported in the Gothenburg protocol and the Cleveland group protocol which recommended administering tacrolimus starting post-operative day 5 with a starting dose of 1–2 mg twice a day, with a target level of 7–11 ng/mL. Methylprednisolone is tapered from 50 mg IV every 6 h to 20 mg IV every 12 h; then prednisone 20 mg is given per os. The tapering protocol considers how long the patient has been on steroid therapy (3 weeks-6 months).

##### 4.1.2.2 Preconception counseling and timing of pregnancy

Pregnancy in uterine VCA recipients should only be pursued once allograft function is stable. Extrapolating from solid organ transplantation, the Society for Maternal-Fetal Medicine recommends deferring pregnancy for at least 1 year after solid organ transplant (and 2 years in lung transplantation). This approach minimizes the risks of allograft rejection, infection, and complications associated with high-dose immunosuppression used in the early post-transplant period (12).

Pregnancy should also be avoided for at least 6 weeks after discontinuing MMF and transitioning to AZA. MMF is teratogenic, and the Society for Maternal-Fetal Medicine, as well as the Kidney Disease: Improving Global Outcomes (KDIGO) guidelines, recommend discontinuing MMF at least 6 weeks before conception and using highly effective contraception during this period (112). Transition to AZA is advised because it has a more favorable safety profile in pregnancy.

##### 4.1.2.3 During pregnancy and until delivery

Once pregnancy is achieved, maintenance immunosuppression must prioritize graft survival while considering fetal safety and physiological changes related to pregnancy in the recipient which may impact the pharmacokinetics and pharmacodynamics of different agents. Hence, all teratogenic drugs, such as MMF, should be discontinued well in advance of embryo transfer or conception.

Maintenance immunosuppression regimens in pregnant patients typically center on combination therapy with CNIs (tacrolimus), AZA, and low-dose corticosteroids (12, 113, 114). This is consistent with studies in pregnant patients after solid organ transplantation or with rheumatologic or autoimmune diseases (8). As some of these agents have been associated with hypertension, hyperglycemia or low birth weight, monitoring blood pressure and thoroughly treating hypertension, maintaining strict glycemic control, monitoring for signs of infection, and assessing the levels of immunosuppressive medications more frequently is critical.

Building on the emerging understanding of pregnancy as a tolerogenic immunologic state, we propose that immunosuppression dosing during gestation be cautiously reduced to align with endogenous mechanisms of maternal-fetal tolerance. Expansion of regulatory T cells, suppression of pro-inflammatory T-cell responses, and the development of microchimerism together suggest that pregnancy may provide a biologic window during which pharmacologic immunosuppression can be carefully reduced without compromising graft stability (109, 110).

However, any such taper must be undertaken with considerable caution. Excessive or poorly timed reduction in immunosuppression could destabilize this tolerogenic balance, precipitate an immunogenic shift, and increase the risk of acute or chronic graft injury, donor-specific antibody formation, or fetal compromise (115). For this reason, reduction of immunosuppressive burden during pregnancy should be individualized and accompanied by close surveillance for early evidence of immune activation. Potential monitoring strategies may include serial assessment of regulatory T-cell profiles, donor-derived cell-free DNA, donor-specific antibodies, and, where feasible, microchimerism assays (116, 117). Together, these approaches may help identify patients in whom physiologic tolerance is being maintained vs. those in whom microchimerism or alloimmune stimulation may signal emerging immunologic risk.

##### 4.1.2.4 Monitoring and delivery planning

Pre-delivery planning for pregnant recipients of uterine VCA requires a multidisciplinary team approach, involving transplant specialists, maternal-fetal medicine, anesthesia, and neonatology, to address the complex medical and surgical needs throughout pregnancy and delivery (118, 119). Patient education, psychosocial support, and strict adherence to immunosuppressive regimens are essential to optimize outcomes and minimize risks of graft rejection and maternal complications.

Structured surveillance during uterine VCA pregnancy should include cervical biopsies at 12–14 and 24–26 weeks' gestation for rejection monitoring, tacrolimus trough level assessment every 4 weeks, high-risk obstetric follow-up every 2–3 weeks, serial doppler ultrasound of uterine and umbilical arteries, and fetal growth assessments every 4–6 weeks following the anatomical survey (118). Given the elevated incidence of preeclampsia and prematurity reported in uterine transplant cohorts, planned cesarean delivery at 37–38 weeks is standard practice. Comprehensive laboratory monitoring, including serum creatinine, liver enzymes, complete blood count, blood glucose, iron studies, and urine albumin-to-creatinine ratio—should be performed every 4–8 weeks throughout pregnancy, with more frequent evaluation if there are concerns for rejection, subtherapeutic immunosuppression, or maternal complications (96, 120). Low-dose aspirin is routinely used for preeclampsia prophylaxis, and serial fetal growth assessments are recommended due to the increased risk of fetal growth restriction (118). Antimicrobial prophylaxis is implemented to reduce infection risk.

##### 4.1.2.5 Postpartum and graft removal

Postpartum management requires shared decision-making regarding graft hysterectomy and the possibility of future pregnancies, given the temporary intent of uterine VCA and the goal of minimizing long-term immunosuppression (118, 121). If childbearing is complete, elective allograft removal is typically performed following delivery.

Although removal of the uterine allograft eliminates the need for continued immunosuppression, withdrawal of therapy must be carefully managed. Corticosteroids should be tapered gradually to avoid acute adrenal insufficiency and systemic complications. Close laboratory monitoring, including platelet counts and inflammatory markers, is recommended during the tapering process. Continued surveillance for infection and delayed rejection phenomena remains important in the early postpartum period (118, 120).

Slow tapering or withdrawal of immunosuppressive therapy is known to result in donor-specific antibody development and subsequent graft fibrosis in solid organ transplant applications. In the immunologic milieu of pregnancy, however, this may represent an opportunity to wean immunosuppression in the midst of a sustained tolerogenic shift. Establishing such a protocol would explore the potential of deferring hysterectomy indefinitely. This option, however, must be weighed against potential contraindications to breastfeeding in the post-partum period.

Importantly, the postpartum phase carries unique physiologic and psychosocial vulnerabilities, including an increased risk of major depressive episodes (122). Multidisciplinary follow-up should therefore address both medical and mental health considerations to ensure safe immunosuppression withdrawal and maternal recovery.

##### 4.1.2.6 Anti-rejection and flares

Return of menstrual cyclicity is typically expected within 90 days following uterine transplantation (8). Delayed onset of menses should prompt evaluation for graft dysfunction or vascular compromise. As uterine allograft does not contain a cutaneous component to monitor and/or biopsy, and rejection may be asymptomatic, recipients undergo routine surveillance with monthly cervical biopsies during the early post-transplant period. Rejection should also be suspected in the presence of abnormal vaginal discharge, irregular bleeding, or loss of established menstrual cyclicity.

The incidence of rejection in pregnant VCA recipients compared to non-pregnant VCA recipients is unknown. However, studies from solid organ transplantation note that pregnancy does not appear to increase the risk of graft rejection, with rates of rejection episodes during pregnancy generally low and comparable to non-pregnant transplant recipients, provided that conception occurs after graft stability is achieved (19). However, these findings cannot be directly extrapolated to VCA due to differences in graft immunogenicity and the lack of VCA-specific data.

Nonetheless, borderline or mild rejection should be managed with increased maintenance immunosuppression medications levels and addition of a low dose of methylprednisolone, whereas moderate to severe rejection should be treated with high doses of methylprednisolone, ATG and AZA.

##### 4.1.2.7 Dosing

Pregnancy-related pharmacokinetic changes frequently necessitate dose adjustments of 20%–25% and close therapeutic drug monitoring every 2–4 weeks to prevent subtherapeutic levels and rejection (12, 19).

### 4.2 Future directions and strategies for uterine VCA pregnancy

#### 4.2.1 Non-invasive monitoring tools

Current rejection surveillance in uterine VCA relies on serial cervical biopsies, which, although effective, are invasive and may carry procedural risks, particularly during pregnancy (120). As such, the development of non-invasive approaches for rejection monitoring represents a critical unmet need in uterine VCA.

Emerging strategies include the identification of uterus-specific immune biomarkers. Elevated expression of markers such as keratin 1, granzyme B, and IL-1β has been reported in association with uterine allograft rejection, suggesting potential utility for earlier diagnosis and more timely therapeutic intervention (123). While preliminary data are promising, validation in larger, prospective cohorts is required.

Donor-derived cell-free DNA (dd-cfDNA) represents another promising genomic biomarker. By quantifying circulating fragments of donor DNA in the recipient's plasma, dd-cfDNA enables detection of allograft injury and has demonstrated clinical utility across solid organ transplantation, including early identification of acute rejection and the development of *de novo* donor-specific antibodies (91, 120, 124). Its application to uterine transplantation could allow for dynamic, minimally invasive graft surveillance throughout pregnancy.

Additional investigational approaches include blood and urine transcriptomic profiling. Gene expression signatures and urinary chemokines, particularly CXCL9 and CXCL10, have shown promise in improving prediction of both subclinical and clinical rejection in kidney transplantation and may offer translational potential for uterine VCA (125).

#### 4.2.2 Immune tolerance and reduced immunosuppression

Induction of immune tolerance is another strategy for prevention of graft rejection and poses an alternative to long term immunosuppression. The concept of immune tolerance suggests that an immune-competent host does not mount an effector response towards donor-specific antigens in the absence of immunosuppressive therapy. The success of tolerance induction, however, is varied in animal studies. The lifetime exposure of humans to various pathogens compared to genetically curated animal models has been associated with resistance to immune tolerance induction (126).

Achieving immune tolerance would be advantageous in the setting of uterine VCA, circumventing both maternal and fetal risks associated with the abovementioned agents. Despite poor overall understanding of this technique, immune tolerance has been described in the setting of several solid organ transplant recipients including liver and kidney, whereby donor hematopoietic stem cells in transplanted tissues were successfully engrafted within the recipient bone marrow without prior myeloablative therapy, establishing hematopoietic chimerism. Hematopoietic chimerism of donor immune cells results in central modulation of the immune response via negative selection in the thymus. Previously documented cases of chimerism in solid organ transplant showed survival of the graft after the cessation of immunosuppressive therapy and return of immune competence (126). In a study of 29 HLA-matched and 22 HLA -haplotype matched kidney transplant recipients, transient mixed chimerism allowed for complete withdrawal or narrowing of immunosuppression with tacrolimus monotherapy, respectively, without rejection nor evidence of graft-vs.-host disease (127). These promising results have sparked numerous pilot studies exploring strategies to achieve hematopoietic chimerism.

The current state of the literature on induction of hematopoietic chimerism spans both animal and human studies and focused on targeted memory T-cell depletion with novel agents including alefacept, siplizumab, and anti-OX40 antibody (128–130). In addition to negligible safety data of these agents, some associated regimens report the use of cyclophosphamide, necessitating sufficient washout period or even precluding their exploration in pregnancy. Importantly, Blein et al. note the fragile nature of tolerogenesis via chimerism, with reports of T-cell mediated rejection in a patient with established immune tolerance following a respiratory infection (131). This foreshadows challenges with achieving immune tolerance in uterine VCA, as pregnancy presents several acute physiological changes that may destabilize immune system quiescence. Beyond stem cell chimerism, immune tolerance regimens are currently being explored through co-transplantation of donor thymus, regulatory T-cell therapy, and biologics (56).

The temporary nature of uterine transplantation provides a unique opportunity to investigate tolerance induction strategies. Cell-based therapies, such as adipose-derived mesenchymal stromal cells (AD-MSCs), bone marrow-derived MSCs, and stromal vascular fraction (SVF), have demonstrated immunomodulatory properties that can prolong VCA survival and potentially induce donor-specific tolerance when combined with short-course immunosuppression (132, 133). Interestingly, regimens in animal models of VCA combining cell-based therapies with lymphodepletion agents and conventional short-course immunosuppressants like tacrolimus consistently induced donor-specific tolerance, with 86% achieving long-term rejection-free survival (134).

Moreover, microparticle-based systems engineered to release Treg-inducing factors (TGF-β1, IL-2, rapamycin) or Treg-recruiting chemokines (CCL22) have achieved indefinite allograft survival and donor-specific tolerance in experimental VCA models without long-term systemic immunosuppression (135, 136).

Although these therapies can improve VCA tolerance by reducing the need for high-dose immunosuppression and averting its side-effects, safety concerns persist since most research has been done in animal models.

##### 4.2.2.1 Precision and personalized immunosuppression

Optimizing immunosuppressive therapy in uterine VCA requires individualized approaches that account for pregnancy-specific pharmacokinetic changes. Physiologically based pharmacokinetic models incorporating pregnancy-induced changes in volume of distribution, CYP3A4 induction, and protein binding can simulate drug exposure and guide dosing regimens across trimesters (137).

Additionally, variation in the CYP3A5 genotype significantly affects tacrolimus metabolism and contributes to inter-individual variability in dose requirements. Incorporating genetic polymorphisms into dosing algorithms could enable personalized regimens that maintain therapeutic levels while minimizing toxicity (46).

Transient mixed chimerism, among other novel strategies for induction of immune tolerance offers a new approach for temporary and/or permanent protection of the allograft while circumventing or significantly lowering both maternal and fetal risks of immunosuppressive therapy. Exploring these strategies in the context of uterine VCA would hold immense implications for the safety of uterine VCA in the treatment of uterine-factor infertility.

Current immunosuppressive regimens in uterine transplantation are largely adapted from kidney transplantation without uterus-specific optimization. Prospective studies comparing different induction agents, maintenance regimens, and tacrolimus formulations are needed to establish evidence-based, pregnancy-specific protocols (7, 8).

### 4.3 Redefining rejection monitoring for uterine VCA during pregnancy

There are reports of uterine VCA rejection during pregnancy, requiring escalation of immunosuppressive agents and/or anti-rejection therapy. Consequent effects on the fetus have been somewhat favourable in these cases, however, exposure to high dose immunosuppressants, including those that are considered safe in pregnancy, can result in both maternal and perinatal complications as discussed elsewhere in this review.

Unlike traditional VCAs where skin biopsies are readily accessible, rejection in uterine VCA is monitored via cervical biopsy and this is currently recommended in our suggested management framework (Figure 1); however, the cervix is histologically distinct from the uterine graft itself. This may result in overdiagnosis of rejection events, wherein the actual uterine graft remains in good health while tissues of higher immunogenicity experience changes, as is the dilemma described in the use of sentinel skin flaps for detection of rejection in organ transplants such as the lung (138). As uterine VCA continues to evolve, clinicians and scientists may consider alternative measures for rejection of the uterine allograft, which may include detection of donor-antibodies or dd-cfDNA.

### 4.4 Long term maternal and child outcomes

In patients on immunomodulatory regimens for UC, combination therapy with AZA, TNFa, anti-integrin, and anti-interleukin 12/23 was found to present low risk to pregnancy with no association between therapy and congenital anomalies or pregnancy outcome. Long term impacts on infant immunity remain unclear, and studies are currently investigating the effects of these drugs on infant reaction to live-vaccination (139). As the number of uterine VCA recipients increase, these regimens may provide new options for immune modulation in the setting of inadequate response to empiric regimens such as the Swedish Protocol.

Given the unique aspects of uterine VCA, long-term effects of temporary immunosuppression on recipients and the developmental outcomes of children exposed *in utero* remain incompletely characterized (120). Dedicated registries and prospective studies are needed to characterize maternal and child outcomes specific to this population.

### 4.5 Additional gaps in the literature

This review explores a breadth of literature relevant to the clinical consideration surrounding immune suppression in the context of uterine transplant pregnancy. In the context of this new and promising treatment modality, understanding of the complex interplay between immunosuppressive agents, VCA, and pregnancy is paramount and several gaps in the collective understanding of the literature remain. In addition to others mentioned in this section, these include the clinical approach to under-response to AZA (and potentially other key drugs) in pregnancy, potential implications of immune suppressing agents on the success of IVF and other fertility interventions for initiation of pregnancy, as well as possible immune privilege conferred by placenta, lymphocyte shifts and physiological changes of pregnancy.

### 4.6 Limitations

This review has several important limitations. First, there is a lack of standardized immunosuppressive protocols specific to uterine transplantation; most, if not all, regimens are adapted from kidney or other solid organ transplant protocols, which may not fully account for the unique pharmacokinetic and immunologic demands of pregnancy and the temporary nature of the uterine graft (8, 140). Other studies are based on the treatment of autoimmune disorder treatment in pregnancy, which does not consider the complex interplay of pregnancy and graft immunology. Moreover, the available clinical data are limited by small sample sizes, heterogeneous patient populations, and short-term follow-up, making it difficult to draw robust conclusions about maternal, fetal, and graft outcomes, as well as long-term safety (7, 141).

Monitoring for rejection during pregnancy relies on invasive cervical biopsies, which are performed infrequently and may miss subclinical rejection; noninvasive diagnostic tools are still under development and not yet validated for routine use (120). There is insufficient evidence regarding the effects of immunosuppressive drugs on fetal development, neonatal immune function, and long-term child outcomes, as most studies report only short-term results and lack comprehensive neurodevelopmental or immunologic follow-up.

Moreover, the risk of pregnancy complications, graft failure, and infection is elevated in this population, but the literature is limited in its ability to stratify risk or provide evidence-based guidance for management, especially in recipients with prior rejection episodes or those desiring repeat pregnancies. Finally, although efforts were made to comprehensively synthesize available literature, selection and publication bias cannot be excluded due to the study's narrative review nature.

## 5 Conclusion

Uterine VCA has demonstrated that pregnancy is feasible in recipients of a temporary uterine graft, offering a novel therapeutic pathway for individuals with uterine-factor infertility. Pregnancy-associated pharmacokinetic changes, however, necessitate close therapeutic drug monitoring and timely dose adjustments to prevent rejection while minimizing maternal and fetal toxicity. In turn, successful outcomes require a carefully structured, stage-based immunosuppressive strategy that spans pre-transplant evaluation, post-transplant stabilization, preconception counseling, pregnancy management, indications for allograft removal, and postpartum care.

Moving forward, the development of non-invasive rejection surveillance tools and precision-guided immunosuppressive strategies, including tolerance induction approaches, may redefine long-term management in uterine VCA. Continued prospective data collection and registry-based collaboration will be essential to establish evidence-based, pregnancy-specific protocols in this evolving field.
